# Identification of a novel *Plasmopara halstedii *elicitor protein combining *de novo *peptide sequencing algorithms and RACE-PCR

**DOI:** 10.1186/1477-5956-8-24

**Published:** 2010-05-10

**Authors:** Stephan Jung, Claudia Fladerer, Frank Braendle, Johannes Madlung, Otmar Spring, Alfred Nordheim

**Affiliations:** 1 Proteome Center Tuebingen, Interfakultaeres Institut fuer Zellbiologie, Universitaet Tuebingen, Tuebingen, Germany; 2 Institut fuer Botanik, Universitaet Hohenheim, Stuttgart, Germany

## Abstract

**Background:**

Often high-quality MS/MS spectra of tryptic peptides do not match to any database entry because of only partially sequenced genomes and therefore, protein identification requires *de novo *peptide sequencing. To achieve protein identification of the economically important but still unsequenced plant pathogenic oomycete *Plasmopara halstedii*, we first evaluated the performance of three different *de novo *peptide sequencing algorithms applied to a protein digests of standard proteins using a quadrupole TOF (QStar Pulsar i).

**Results:**

The performance order of the algorithms was PEAKS online > PepNovo > CompNovo. In summary, PEAKS online correctly predicted 45% of measured peptides for a protein test data set.

All three *de *novo peptide sequencing algorithms were used to identify MS/MS spectra of tryptic peptides of an unknown 57 kDa protein of *P. halstedii*. We found ten *de novo *sequenced peptides that showed homology to a *Phytophthora infestans *protein, a closely related organism of *P. halstedii*. Employing a second complementary approach, verification of peptide prediction and protein identification was performed by creation of degenerate primers for RACE-PCR and led to an ORF of 1,589 bp for a hypothetical phosphoenolpyruvate carboxykinase.

**Conclusions:**

Our study demonstrated that identification of proteins within minute amounts of sample material improved significantly by combining sensitive LC-MS methods with different *de novo *peptide sequencing algorithms. In addition, this is the first study that verified protein prediction from MS data by also employing a second complementary approach, in which RACE-PCR led to identification of a novel elicitor protein in *P. halstedii*.

## Background

Over the last decade, mass spectrometry has evolved as an indispensable tool in protein analysis [1,2]. Recent improvements enable the elucidation of sequence information from limited amounts of protein by usage of MS/MS which is the most reliable way to identify proteins [3]. However, MS analysis of proteolytic peptides generates thousands of MS/MS spectra in a single experiment [4]. Matching these spectra to peptides manually would be a near impossible task. Consequently, computational solutions were generated and today, automated peptide identification is performed by database search algorithms, the most popular being Sequest [5] and Mascot [6].

Although search algorithms perform an automated search for peptide identification and allow a high-throughput mode for modern proteomics laboratories, database searches do not solve all problems. Basic conditions for automated database searches are an accurately sequenced genome or highly homologue genomes as well as annotation of all protein coding genes or in some cases EST. Because many alternatively spliced genes exist, the latter condition is rarely fulfilled. In addition, search algorithms fail to identify some peptides because of limitations in the searches like artificially modified amino acids, single nucleotide polymorphisms and unknown post-translational modifications [7]. Hence, many high-quality MS/MS spectra of proteolytic peptides do not match to any database entry.

*De novo *peptide sequencing overcomes one of the above mentioned problems as this procedure allows the reconstruction of the original peptide from the MS spectrum without knowledge of the genome sequence or even of the organism from which the peptides are derived. The aim of *de novo *sequencing is to obtain the correct amino acid sequence of the peptide irrespective of the nucleic acid sequence from the MS/MS spectrum.

Since manual *de novo *sequencing of peptides is time-consuming many *de novo *sequencing algorithms have been developed to perform computational identifications automatically. Most algorithms employ a graph-theoretical approach by representing the spectrum with a "spectrum graph" [8]. Popular examples for this approach are Lutefisk [9,10] and PepNovo [11]. Different approaches are used by PEAKS online [12,13], NovoHMM [14], PILOT [15] and CompNovo [16]. For further information, a review of several common *de novo *algorithms is given elsewhere [17,18].

Although all articles presenting a new *de novo *sequencing algorithm implement a performance comparison of different algorithms, to our knowledge, only two studies exist from independent laboratories which have systematically evaluated multiple algorithms [19,20]. None of these studies included *de novo *algorithms supplied by OpenMS [21,22] which could potentially have widespread use due to their association with many other proteomics tools like database searching and false discovery rate estimation.

In addition to different *de novo *sequencing algorithm approaches, many user-defined parameters on the MS instrument influence their outcome. For example, the deduction of amino acid sequences from MS/MS spectra is dependent on the quality of spectra data, since incomplete ion series of peptides lead to low-quality MS/MS data. The typical high noise level of MS/MS measurements in high-throughput experiments limits the performance of *de novo *sequencing tools and strongly favours probabilistic models for the data analysis [23,24]. Also, mass accuracy of the mass spectrometer used for generation of MS/MS fragments is an important factor for differentiation between amino acids with little mass difference [25].

Another pitfall in *de novo *sequencing is the identification of the protein to which these sequences belong. Conventional BLAST searches fail in most cases because these engines are optimized to identify similarities between fairly long protein sequences and do not cope well with short sequences and isobaric amino acids of same masses (e.g. I/L and Q/K). Thus, those sequences are normally applied for error-tolerant sequence-similarity searches by engines like MS driven BLAST (MS Blast) [26], FASTS [27], OpenSea [28,29] or SPIDER [30].

These approaches have been successfully applied to varies proteomic studies of different organisms including green algae [31], yeast [26], monkey [32] and human [28]. To our knowledge, the oomycete *Plasmopara halstedii*, an economically important pathogen causing downy mildew in sunflower, was not studied in-depth and there is currently only limited knowledge regarding proteins from this unsequenced pathogen that are involved as signals in the host resistance. This is mostly due to the limitations arising from the biotrophic nature of the parasite, which prohibits cultivation apart from in the living host, and consequently, only minute amounts of material can be obtained for experimental investigation.

Here, we present a study divided in two parts. In the first part we evaluated the performance of three different *de novo *algorithms: CompNovo, PEAKS online, and PepNovo using a quadrupole TOF (QTOF). In the second part we applied this *de *novo sequencing workflow to identify an unknown putative elicitor protein of *Plasmopara halstedii *by database searching and sequence-similarity searches. This protein identification was afterwards verified by RACE-PCR.

## Results

### Performance evaluation of CompNovo, PepNovo and Peaks online utilizing a protein standard

An initial literature review was performed in order to assess existing performance comparisons of *de novo *sequencing algorithms. The result of this search is summarized in additional file 1 table S1. While PEAKS online seems to be superior for QTOF data, PepNovo leads the field for *de novo *sequencing with IT data. The newly developed PILOT algorithm claims to be superior for data acquired by both types of analyzers but was still not publicly available at the time of writing. In our study, we concentrated on two of the previously known best algorithms for QTOF data: PEAKS online and PepNovo. We evaluated their performance in comparison to CompNovo, a new *de *novo sequencing algorithm developed for CID and ETD spectra.

Tryptic digests of a protein test set (alcohol dehydrogenase 1, cytochrome C, glycogen phosphorylase b, enolase 1, BSA, haemoglobin subunit a and subunit b, L-lactate dehydrogenase, alpha casein 1 and 2) were measured by QSTAR, and acquired MS/MS spectra were analysed by Mascot database searching. By applying a false-discovery rate of 1%, a total of 78 peptides were identified consisting of one singly, 62 doubly and 15 triply charged peptides. The same data was processed with *de novo *sequencing algorithms. The top-ranked sequence reported from each program was extracted and compared to Mascot results (additional file 1 table S4). The raw data of the protein test set were converted to the mascot generic file (mgf-format). All processed data are included in additional file 2.

Different evaluation measures for performance comparison were used. First, the overall peptide prediction accuracy was considered. In terms of prediction accuracy for correct sequence annotation (peptide length of minimum ten amino acids), PEAKS online outperforms all other programs with an identification rate of 45% followed by PepNovo (18%), and CompNovo (14%) for QSTAR data (table 1). The corresponding *de novo *sequences are summarised for each algorithm (additional file 1 table S4).

**Table 1:** Prediction accuracy of test data set

	x ≥ 3	x ≥ 4	x ≥ 5	x ≥ 6	x ≥ 7	x ≥ 8	x ≥ 9	x ≥ 10
Number of peptides with length ≥ x	78	78	78	78	78	70	57	44

CompNovo 0.9	52 (67%)	47 (60%)	39 (50%)	37 (47%)	31 (40%)	20 (29%)	12 (21%)	6 (14%)

PEAKS Online	75 (96%)	69 (88%)	64 (82%)	58 (74%)	52 (67%)	40 (57%)	29 (51%)	20 (45%)

PepNovo	61 (78%)	55 (71%)	45 (58%)	41 (53%)	31 (40%)	20 (29%)	10 (18%)	8 (18%)

The table shows the accurate prediction accuracy of the algorithms for QSTAR obtained spectra in relation to different peptide lengths (shown as x).

**Figure 1:**
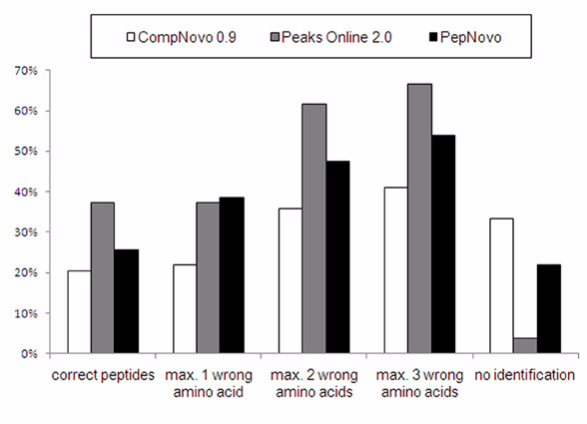
**Prediction accuracy of QSTAR data**. Number of correct peptides in percent with up to three false amino acid assignments allowed and no identification are shown.

Second, it was considered that most *de novo *sequencing algorithms incorrectly assign isobaric residues (additional file 1 table S3) that could not be differentiated by the QSTAR. Thus, up to three incorrect amino acid assignments were allowed, and this resulted in an improvement of prediction accuracy for all algorithms: PEAKS online (67%) > PepNovo (54%) > CompNovo (41%) (fig. 1).

Finally, the average rate of correct amino acid prediction per peptide was calculated for each algorithm by dividing the correctly predicted amino acids for all peptides through total predicted amino acids. PEAKS online was superior with an average rate of 71% of correctly predicted amino acids per peptide followed by PepNovo (59%), and CompNovo (54%) (additional file 1 table S5).

### Identification and *de novo *sequencing of an elicitor protein of *P. halstedii*

**Figure 2:**
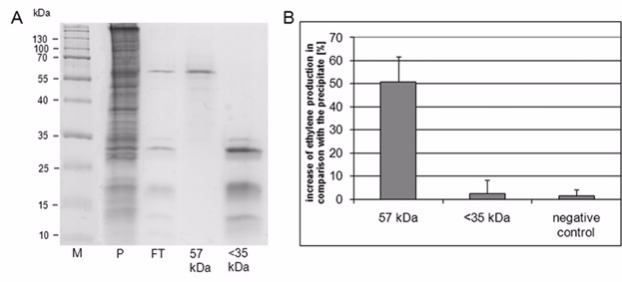
**Elicitor activity of identified *P. halstedii *57 kDa protein**. Figure (A) shows a 13% SDS-PAGE of the *P. halstedii *protein purification. Lane 2 (P) displays the ammonium sulphate precipitate, lane 3 (FT) shows the purified proteins of the flow-through, lane 4 (57 kDa) displays the gel extraction section from lane 3 at M_r _50-70 kDa and lane 5 (<35 kDa) shows the gel extraction section from lane 3 at M_r _below 35 kDa. The diagram (B) shows the effect of the extracted proteins of the flow-through to the ethylene induction of sunflower leave-disks 3 h after infiltration. The three columns display the activity of the gel extraction section of the 57 kDa protein (lane 4, 57 kDa), the gel extraction section of proteins <35 kDa (lane 5, <35 kDa) and the negative control. M represents marker (n = 3).

The putative elicitor protein, which induced ethylene release within minutes, was isolated using ammonium sulphate precipitation, ion-exchange chromatography, SDS-PAGE and gel extraction (fig. 2A). Bioassay-guided fractionation of cellular extracts from sporangia of *P. halstedii *led to the isolation of a 57 kDa polypeptide, which showed elicitor activity in sunflower *H. annuus*, the host plant of this biotrophic oomycete (fig. 2b). Tryptic peptides from the 1D-SDS-PAGE protein band with elicitor activity (fig. 2A, lane 3 (57 kDa)) were measured on QSTAR to obtain a fragmentation pattern. The raw data of the *P. halstedii *sample was converted to the mascot generic file (mgf-format) and was included in additional file 2.

Automatic Mascot database searching of these MS data using SwissProt database (version of 2009-03-19) resulted in five proteins. Four of these five proteins were only identified by a single peptide, a so-called "one-hit wonder". A fifth protein was identified only by two peptides (additional file 1 table S6).

When checking relationship in a phylogenic tree for a house-keeping enzyme like cytochrome oxydase II between *P. halstedii *and other organisms (additional file 1 fig. S6) it was shown that cytochrome oxydase II is more related to a human homolog than any bacterial protein. However, in our database search of the *P. halstedii *MS data we identified only bacterial proteins.

We employed a *de novo *peptide sequencing approach as described before, because (i) "one-hit wonders" are not reliable at all, (ii) identification of a protein with only two peptides is still inadequately verified for solid scientific research, and (iii) all automatically identified proteins are from bacteria which have little relationship to the oomycete *P. halstedii *according to the phylogenetic tree.

As previously suggested [32,33], a high threshold of 90 as confidence score for *de novo *sequences using PEAKS online was set. Using this confidence score for PEAKS online and after removing contaminations such as spectra from autolytic trypsin peptides (4 peptides), six peptide candidates were identified. For PepNovo, we also set the threshold to 90 that resulted in 17 peptide candidates. 13 of these 17 peptides are additional peptides, which were not ranked with a threshold above 90 by PEAKS online. This resulted in a total of 19 candidate peptides, of which six peptides could not be verified manually and were discarded. Of these remaining 13 peptide candidates four candidates showed identical masses and similar *de *novo sequence (m/z 535.7 and 535.2; 638.8 and 638.3). Of these four candidates with similar mass and sequences, we discarded the candidates with lower confidence scores and only kept the higher scored *de *novo sequences. One peptide candidate showed the same *de *novo sequence as another candidate with the only difference in having a missed cleavage site. We did not include this candidate (m/z 489.7) in our list, but used it for verification of the protein sequence. The remaining ten peptide candidates (m/z 425.7, 494.7, 517.7, 535.7, 542.7, 554.7, 575.2, 638.2, 654.8 and 682.3) are shown in figs. Four and additional file 1 fig. S8, as well as in table 2.

**Table 2:** De novo sequenced peptide candidates of P. halstedii predicted by PEAKS online, PepNovo, and CompNovo

m/z	z	Sequence	de novo score	Position (consensus [%])
425.7	2	YADLLQK	94	468 - 474 (100)
		
		**YADLLKK**	111	468 - 474 (100)
		
		*YANNLQK*	0.223	468 - 474 (71)

494.7	2	TTLSADQPR	14	302 - 310 (78)
		
		**TTLSADS [313.151]**	98	302 - 308 (86)
		
		TTLSADQPR	0.029	302 - 310 (78)

517.7	2	NPFGM^Ox^EVPK	37	526 - 535 (80)
		
		**[96.894]NFGFEPVK**	102	528 - 535 (75)
		
		*PNM*^*Ox*^*GTTGVNK*	0.130	486 - 499 (43)

535.7	2	DGTYTLDTGK	99	103 - 112 (90)
		
		**DGTYTMQ [274.035]**	95	103 - 109 (57)
		
		*DGTYTLDTGK*	0.080	103 - 112 (90)

542.7	2	LPDFYNTSK	99	368 - 376 (89)
		
		**LPDM**^Ox^**YNTSK**	96	368 - 376 (89)
		
		*DGTYTLDTGK*	0.080	103 - 112 (90)

554.7	2	YLVDEAPSSK	100	122 - 131 (90)
		
		**YLVDEAP [321.163]**	93	122 - 128 (86)
		
		YLVDEASRR	0.122	122 - 130 (56)

575.2	2	DPNM^Ox^GFEVPK	19	526 - 535 (100)
		
		**[212.052 ]NFGM**^Ox^**LDPK**	90	528 - 535 (75)
		
		*DKDDTTMVPK*	0.120	553 - 562 (40)

638.3	2	C^CAMe^ALDALGNGGSLK	34	508 - 518 (62)
		
		**[231.052]LDALDLGD [232.049]**	108	510 - 517 (63)
		
		*DDNDALMRLGR*	0.089	286 - 296 (27)

654.8	2	LGSLPENVRAPR	88	537 - 548 (83)
		
		**LGSLPENVLN [272.154]**	93	537 - 546 (100)
		
		*LGSLPENQVVPR*	0.055	537 - 548 (75)

682.3	2	WLLENVFVDTK	87	355 - 365 (91)
		
		**[299.126]LENVM**^Ox^**TLTK**	96	357 - 365 (78)
		
		*NNALENVM*^*Ox*^*VDTK*	0.118	354 - 365 (75)

The table presents the peptide candidates predicted by PEAKS online, PepNovo (bold), and CompNovo (italic). Position of peptide candidates is shown in accordance to their position to translated ORF of *P. halstedii *protein (numbers in brackets represent consensus to alignment in percent).

Applying all *de novo *sequenced peptides from the different algorithms to a similarity-sequence search using BLAST and MS BLAST did again only result in similarity of low scored peptide hits of bacteria (additional file 1 figs. S8 and S9).

**Figure 3:**
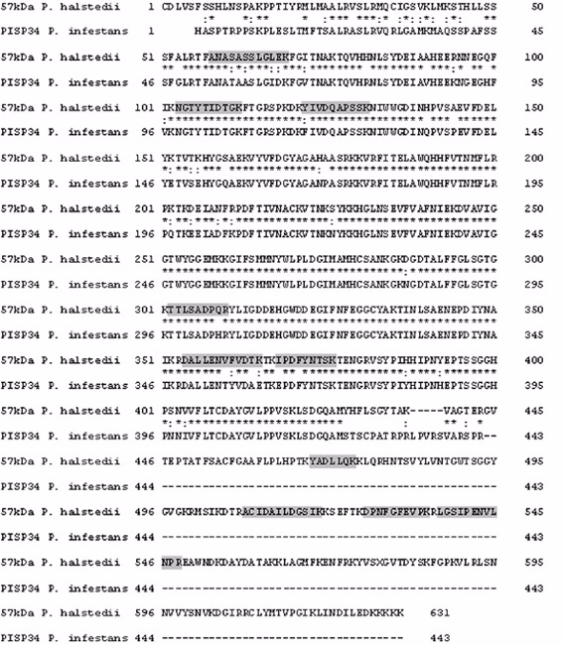
**Homology alignment of *P. infestans *(putative phosphoenolpyruvate carboxykinase; PISP34) and *P. halstedii *57 kDa protein**. Partial alignment of PISP34 protein of *P. infestans *to the 57 kDa elicitor protein of *P. halstedii *is shown. Identity between both proteins amounts to 359 of 636 amino acids (56.4%). Asterisks (*) denotes homology and colon (**:**) denotes similarity of amino acids. Boxed letters (grey) mark *de novo *sequenced peptide candidates as shown in table 2.

Degenerate oligonucleotides were constructed from candidate peptides in order to identify the genomic sequence encoding for the 57 kDa polypeptide. PCR with the primer pair F1 + R1 using genomic DNA led to an amplicon approximately 700 bp in length. The PCR product was identified by direct sequencing using the primer pair F2 + R2 (fig. 3; internal amplicon). The 3'-terminus and 5'-terminus were elucidated by RACE-PCR, using RNA from sporangia as a template for full-length cDNA-synthesis.

**Figure 4:**
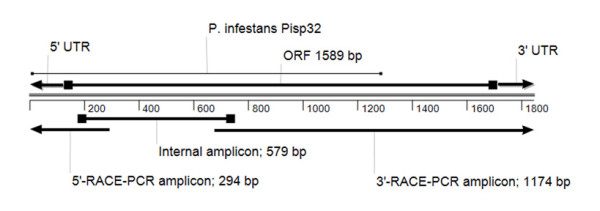
**Scheme of PCR experiments**. The alignment shows the three amplicons, which led to the ORF and untranslated regions (UTRs) of the 57 kDa protein of *P. halstedii*. The internal amplicon was revealed with the primer pairs F1+R1 and F2 + R2 (nested PCR). The 3'-RACE-PCR amplicon was revealed with the gene-specific primer PhE-F1 and PhE-F2 (semi-nested PCR). The 5'-RACE-PCR amplicon was revealed with the gene-specific primer PhE-R1, PhE-R2 (semi-nested PCR) and PhE-R3 (semi-nested PCR). In all RACE experiments the provided adaptor primer of the SMART™ RACE cDNA amplification kit was used.

Sequence comparison of the two RACE-PCR amplicons with the internal amplicon showed 100% identity in the overlapping parts. In addition to this, the 3'-RACE-PCR amplicon contained a poly-A motif at the 3'-terminus. The alignment of the three amplicons resulted in an ORF of 1589 base pairs (fig. 4). The translated ORF showed 56% identity to a putative phosphoenolpyruvate carboxykinase (PISP34) of *Phytophthora infestans*, a closely related organism of *P. halstedii *(fig. 3).

Successful DNA sequencing of the complete ORF of the elicitor protein enabled us to verify additional *de novo *sequenced peptides which were below our chosen threshold of 90 and which would further confirm the identified protein. Therefore, we applied a confidence score of 80 for PEAKS online and PepNovo and compared these *de novo *sequenced peptides with the amino acid sequence of the translated ORF. This led to two additional peptides being predicted by PEAKS online and 16 more peptides by PepNovo. After combining both predictions and discarding all peptide candidates which could not be verified manually the total number of completely new peptide candidates was seven. We discarded candidates, which annotated for the same peptide mass and sequence and resulted in a total of five new candidates (m/z 495.4, 574.2, 575.3, 655.3, and 675.8) (see additional file 1 table S7, dark grey marked). Four of these five additional peptides showed at least 75% homology to the translated ORF of *P. halstedii *(m/z 574.5 showed only 50% homology).

To summarize our results, all predicted peptides showed high homology to the elicitor protein, and four peptides showed complete identity (fig. 3; tabs. 2 and additional file 1 table S7). With the exception of peptides m/z 574.2 and 517.7 all peptides fitted perfectly in the cleavage pattern of trypsin. An arginine or lysine is positioned at the N-terminus before each peptide candidate and every peptide candidate ends with a lysine or arginine, which decreases the possibility of a false sequence annotation and therefore, strengthens the probability of a true tryptic peptide (fig. 3). Exactly the same sequence of m/z 517.7 was also annotated in m/z 575.2 that in this peptide perfectly fits into the cleavage pattern of trypsin.

In conclusion, using a combination of PEAKS online, PepNovo and CompNovo led to successful identification of ten different *de novo *sequenced peptides. All predicted peptides were verified by RACE-PCR.

## Discussion

The aim of the present study was to identify an elicitor protein of the unsequenced pathogenic taxon *P. halstedii *where automatic database searches of MS/MS spectra would otherwise fail. To reach this goal, the performance of three *de novo *sequencing algorithms (PepNovo, PEAKS online, and CompNovo) was evaluated. While PEAKS online was reported to be superior in evaluation of QTOF spectra [19] and PepNovo in LIT spectra [19,20], CompNovo had been thoroughly tested in ETD/CID spectra [16].

Three criteria were selected for evaluation of performance quality of *de novo *sequencing algorithms: (i) exact prediction accuracy, (ii) prediction accuracy with a maximum tolerance of up to three incorrect amino acid assignments, and (iii) average rate of correctly predicted amino acids per peptide.

Concerning all criteria, PEAKS online outperformed the other algorithms in each category. Performance order was PEAKS online > PepNovo > CompNovo (fig. 1, table 1).

During the performance evaluation of the protein test data set it was noted that CompNovo only succeeded in 33% (5/15) to predict triply charged ions with 12% of correctly identified amino acids. This value seems to be much lower than the reported 33.5% of correctly identified amino acids of 134 triply charged ions CID/ETD spectra [16] which could be due to the lower number of 15 deployed triply charged ions spectra in our study. In the same study it was shown that PepNovo performed inferior for triply charged ions with an identification rate of 19.5%. However, we showed that PepNovo performed much better with our test data set (49% of correctly identified amino acids in triply charged ions) and that Peaks online performed superior to both algorithms by correctly identifying 57% amino acids in triply charged ions.

We also noted that the scoring system for CompNovo predictions is unreliable since most of the correctly predicted sequences do have very low scores with CompNovo in comparison to Peaks Online and PepNovo (additional file 1 table S4).

Our study showed that all tested *de novo *algorithms failed to reach a 50% threshold of exact peptide sequence identification (fig. 1) which is in line with other published reports [19]. Recently, employment of PepNovo and NovoHMM with stringent filtering criteria succeeded in high-throughput *de novo *sequencing of peptides of spinach chloroplast, bell pepper chromoplast and Cassave leave and root proteome [34].

A completely different solution for *de novo *sequencing is introduced by the two-stage algorithm PILOT [15], which integrates an integer linear optimisation approach. PILOT has been shown to generate superior prediction accuracy for QTOF data (72%) [15]. Unfortunately, the algorithm was not publicly available at the time of this study.

Based on this performance evaluation we analysed tryptic peptides of an unknown 57 kDa protein of *P. halstedii*. Resulting MS/MS spectra were *de novo *sequenced by all three *de novo *sequencing algorithms. PEAKS online reported eight peptides and PepNovo reported 18 peptides with a high probability score (>80). Combining predictions of both algorithms, a total number of ten unique peptides with high probabilities were identified. Database searches by BLAST and sequence-similarity searches with MS BLAST did result in low scored hits of bacterial peptides (additional file 1 figs. S8 and S9). Verification of peptide prediction and protein identification by degenerate primers for RACE-PCR led to an ORF of 1,589 bp. The translated ORF showed 56% identity to a hypothetical phosphoenolpyruvate carboxykinase (PISP34) of *P. infestans*.

Here, we have shown that the *de novo *sequencing algorithms PEAKS online, PepNovo, and CompNovo result in high prediction accuracy, in this study for Applied Biosystems QTOF data. The combination of all three algorithms improved confidence in the reliability of predicted sequences and increased the total number of predicted peptides as PepNovo and Peaks online ranked different peptides with highest scores. Therefore, using the combined *de novo *peptide sequencing algorithm workflow presented in this study would result in a reduced number of false-positives for high-throughput *de novo *sequencing experiments. In general, PEAKS online amino acid sequence prediction was more accurate (83%) than PepNovo (79%) and CompNovo (56%) prediction for the *P. halstedii *protein. For example, sequence accuracy of PepNovo for m/z 655.3 was 67% with a confidence score of 88 whereas PEAKS online marked the same peptide with a low confidence score of 24 but resulted in higher sequence prediction accuracy (75%). Therefore, we suggest using PEAKS online for prediction of amino acid sequence, using PepNovo as a filter unit of confidence score, and CompNovo for verification of amino acid sequence. That would result in a list of predicted peptides with a high confidence score generated by PEAKS online. The list can then be expanded by additional peptides, which were marked with a high confidence score above 80 in PepNovo. For these additional peptides, the user should utilize the sequences of PEAKS online instead of PepNovo as prediction results.

## Conclusions

Our study demonstrated that identification of proteins within minute amounts of sample material improved significantly by combining sensitive LC-MS methods with *de novo *peptide sequencing. In addition, this is the first study that verified protein prediction from MS data by also employing a second complementary approach, in which RACE-PCR led to identification of a novel elicitor protein in *P. halstedii*. This workflow is likely to be of great economical interest for further identification of plant elicitors, and very useful for studies with low sample amount where Edman sequencing, with its demand for higher quantities, would fail. It also offers an alternative for organisms where error-tolerant database searches and sequence-similarity searches fail to succeed because of low homology to closely related organisms.

## Methods

### Material

The protein test sets were purchased from different companies. Tryptic digests of serum albumin (bovine) and cytochrome c (horse) were purchased from Bruker Daltonics (Bremen, Germany). A mixture (MassPrep digestion standard 1) containing tryptic digests of alcohol dehydrogenase 1 (yeast), glycogen phosphorylase b (rabbit), enolase 1 (yeast) and BSA (bovine) were purchased from Waters (Eschborn, Germany). Haemoglobin subunit a and subunit b (bovine), L-lactate dehydrogenase (rabbit), alpha casein 1 and 2 (bovine) were purchased from Sigma-Aldrich (Munich, Germany). Protein amount is specified in the additional file 1 table S2. ACN, TFA, and formic acid (FA) were purchased from Merck (Darmstadt, Germany), and all other chemicals were purchased from Sigma-Aldrich.

### In solution digest

The protein sample was dissolved in denaturation buffer (6 M urea, 2 M thiourea in 10 mM HEPES buffer) at a final concentration of 1-2 μg protein/μl. 1 M dithiothreitol in 50 mM ammonium bicarbonate was added to the sample to a final concentration of 1 mM DTT and incubated for one hour at room temperature. Afterwards, alkylation buffer (550 mM iodoacetamide in 50 mM ammonium bicarbonate) was added to the sample to a final concentration of 5.5 mM iodoacetamide and incubated for one hour at room temperature in the dark. 1 μg of lysyl endopeptidase LysC (Waco, Japan) per 100 μg protein was added and followed by an incubation for 3 hours at room temperature. The sample was diluted with 4 sample volumes of 20 mM ammonium bicarbonate. One μg trypsin (Promega, Mannheim, Germany) per 100 μg sample protein was added, and samples were incubated overnight at room temperature. Digested proteins were stored at -20°C.

### LC/MS analysis

Peptides from tryptic protein digests were separated on a Dionex Ultimate nanoLC-System (Idstein, Germany) coupled to a QTOF MS (QStar Pulsar i, Applied Biosystems, Darmstadt, Germany). In the following, the QStar Pulsar i MS is defined as QSTAR. LC separation prior to QSTAR acquisition was performed as described before [35].

### Protein identification from MS data

Protein identification was performed using OpenMS (version 1.6) in combination with the Mascot database algorithm (version 2.2; MatrixScience, London) [6].

The protein test sets were identified using a sequence database including all 9,320 protein sequences from *S. cellulosum *of Uni-ProtKB/Swiss-Prot release 15.4/57.4, 86 protein sequences of trypsin and keratin and the protein test set sequences (BSA (bovine); cytochrome c (horse); alcohol dehydrogenase 1 (yeast); glycogen phosphorylase b (rabbit); enolase 1 (yeast); haemoglobin subunit a and subunit b (bovine); L-lactate dehydrogenase (rabbit); alpha casein 1 and 2 (bovine)). In addition, the database contained reversed sequences of all proteins appended to the original *S. cellulosum *database including the contamination proteins and the protein test set sequences to allow a maximum false discovery rate (FDR) of 1%. The *P. halstedii *data was processed using a SwissProt database (version of 2008-12-09).

In both cases, the following parameters were chosen for database search: cysteine carbamidomethylation was included as fixed modification and methionine oxidation was included as variable modification and up to one missed cleavage was allowed during the search runs. The peptide tolerance and MS/MS tolerance was set to 0.3 Da (see additional file 1).

### *De novo *sequencing of peptides

For all *de novo *algorithms, methionine oxidation was selected as variable modification and carbamidomethylation of cysteines as fixed modification. QSTAR data were *de *novo sequenced using peptide tolerance and MS/MS tolerance of 0.3 Da. A maximum of one missed-cleavage was allowed. All data were searched for tryptic peptides (see additional file 1 information).

### Evaluation of *de novo *sequences

The predicted and correctly identified sequences are compared from the left to the right and an amino acid of the predicted peptide sequence is counted as correct if the corresponding amino acid in the correctly identified sequence is identical. For evaluation purposes, only peptides with a minimum of three consecutive correctly identified amino acids were taken into account for determining subsequence length. Due to low mass accuracy the mass spectrometer used in this study is not suitable to differentiate between amino acids with isobaric masses (K/Q, I/L, and F/oxidized M). In such cases, identification of an isobaric amino acid was regarded as a correct prediction. However, if detected masses can either correspond to one large amino acid or two smaller ones that together have the same mass (e.g. W/EG), this was regarded as an incorrect prediction.

Prediction accuracy is defined as the number of peptides with correct amino acid prediction divided through the number of total identified peptides.

### Plant material/*P. halstedii *material

The sunflower *Helianthus annuus cv. Giganteus *(Ernst Benary Samenzucht, Hann. Muenden, Germany), which is highly susceptible to all known *P. halstedii *strains, was used as plant substrate. Plants were cultivated in heat-sterilized soil at 16°C, 80% relative humidity and 14 h light/day prior to and post inoculation with the pathogen. *P. halstedii *was maintained on sunflower seedlings using the whole seedling inoculation technique [36] under cultivation conditions described earlier [37]. Fresh sporangia were washed from cotyledons of infected plants with deionised water and were then used for infection, subsequent DNA and protein extraction.

### Protein isolation from sporangia

5 mg of sporangia were homogenized in a mixer mill (Retsch, Haan, Germany) and suspended in 25 mM Tris/HCl (pH8). After removing insoluble material, proteins were purified by ammonium sulphate precipitation (80% (v/w)) followed by desalting using ultrafiltration spin columns (Vivaspin 500, 10,000 MWCO, Sartorius, Goettingen, Germany). After desalting, 25 μl aliquot were withdrawn from the sporangia protein solution and diluted with 25 mM Tris/HCl (pH8). Ion-exchange chromatography was performed using a spin column system equipped with strong basic anion exchanger (VivaPure IEX Mini H; functional group: Quaternary ammonium; buffer. 25 m*M *Tris/HCl (pH8); Sartorius, Goettingen, Germany), according to manual.

Sporangia proteins were separated on 13% acrylamide slab gels in the discontinuous Tris-glycine system described by Laemmli [29]. Prior to electrophoresis, samples were mixed with a loading dye (Roti-Load 1, Carl Roth, Karlsruhe, Germany) (4:1 v/v) and briefly heated at 90°C. PAGE in Tris-glycine buffer (pH8.6) containing 0.1% SDS was carried out at 25 mA and gels were stained (0.25% Coomassie brilliant blue R-250, 7% acetic acid and 30% methanol). A protein standard (PageRuler™ Plus Prestained Protein Ladder, MBI Fermentas, St. Leon-Rot, Germany) was used for estimating sporangia protein molecular weights. In-gel digestion of target protein was performed as described earlier [38].

### Elicitor induced ethylene production in sunflower

Leaf-disks of three-week-old plants were infiltrated through the abaxial surface with probe solutions. As controls, leaf-disks were similarly treated with water. Infiltrated leaf-disks were transferred into gas-proof glass vials equipped with a septum and further incubated at room temperature for 3 h in the dark. 400 μl from the headspace were taken out with a gas-proofed syringe. The plant stress hormone ethylene was detected using gas chromatography (GC321, HNU Systems. Inc., Newton, Mass., USA, equipped with steel column (packing material: Porapak^® ^80-100 m) and photoionisation detector).

### Evaluation of internal DNA sequences of total nucleic acid from sporangia

For the isolation of total DNA, 5 mg of sporangia was suspended in 800 μL lysis buffer (50 mM NaCl, 10 mM Tris, 20 mM EDTA, pH7.5) and dispersed using an Ultra-Turrax T-8 instrument (IKA-Labortechnik, Stauffen, Germany). DNA was extracted using the GenElute plant mini kit (Sigma Aldrich, Munich, Germany), according to manual. RNA was extracted using a plant Aurum total RNA mini kit (Biorad, Munich, Germany), following the manufacturer's instructions.

To identify internal DNA sequences encoding for the 57 kDa polypeptide the following primers were designed:

Forward primer F1 (5'-TN GGN CTN GAR AAR TTY MGN AT-3') targeted the LGLEKFRI motif. Forward primer F2 (5'-TTY MGN ATH GAY AAY GCN AAR AC-3') targeted the FRIDNAKT motif. Reverse primer R1 (5'-YTT NCC NGT NCC NGW NAR NCC-3') targeted the GLSGTGK motif. Reverse primer R2 (5'-CCR AAR AAN ARN GCN GTR TCN CC-3') targeted the DGDTALFFG motif. Primer pair F1 and R1 was expected to give a PCR product approximately 700 bp in length. Primer pair F2 + R2 was used as nested primer and primer for direct sequencing.

PCR-amplifications were performed with 100 ng of genomic DNA in 25 μL reactions. Each reaction contained 10 mM Tris-HCl (pH8.8), 50 mM KCl, 5 pmol of each dNTP, 1.25 mM MgCl2, and 1U *Taq *polymerase (MBI Fermentas, St. Leon-Rot, Germany). PCR (35 cycles) was carried out in a thermocycler (Eppendorf, Hamburg) under the following conditions: 30s denaturation at 94°C, 60s annealing at 50°C, and 50s strand synthesis at 72°C. Initial denaturation was conducted at 94°C for 5 min and a final extension for 10 min at 72°C. Amplification products were resolved by gel electrophoresis using a 1.5% agarose gel stained with ethidium bromide and photographed under UV illumination.

### RACE-PCR

The 3'- and 5'-end of the gene was elucidated by RACE-PCR using the SMART™ RACE cDNA amplification kit (Clontech, Heidelberg, Germany), according to manual.

Five oligonucleotide primers targeted to the revealed internal nucleic acid sequence and the kit-included adaptor were used for amplification and sequencing. Forward primer PhE-F1 (5'-GACGTGGCTGTTATTGGTGGTAC-3') and PhE-F2 (5'-GGTGGTACATGGTATGGAGGAG-3') were used as gene-specific primer in the 3'-RACE-PCR. Primer pair PhE-F1 + adaptor were used to produce the first amplicons followed by a semi-nested PCR with the primer pair PhE-F2 + adaptor. Reverse Primer PhE-R1 (5'-GTACCACCAATAACAGCCACGTC-3'), PhE-R2 (5'-CGAACGGCCCGTAAATTTGCCAGT-3') and PhE-R3 (5'-CGTGAGCAGCAATTTCATCGTAGC-3') were used as gene-specific primer in the 5'-RACE-PCR. Primer pair PhE-R1 + adaptor were used to produce the first amplicons followed by two rounds of semi-nested PCR with the primer pair PhE-F2 + adaptor and PhE-F3 + adaptor.

All oligonucleotide primers were designed using the FastPCR software version 5.2.2 (available at http://www.biocenter.helsinki.fi/bi/programs/fastpcr.htm).

## Abbreviations

FA: formic acid; LIT: linear ion trap; RACE-PCR: rapid amplification of cDNA-ends using polymerase chain reaction; PTM: post-translational modification; QTOF: quadrupole time-of-flight

## Competing interests

The authors declare that they have no competing interests.

## Authors' contributions

SJ conceived, designed and coordinated the study and performed *de novo *sequencing. CF statistically evaluated prediction accuracy. FB provided the *P. halstedii *sample and performed RACE-PCR. SJ and JM performed MS measurement. OS supervised *P. halstedii *study. AN participated in its design and helped to draft the manuscript. All authors read and approved the final manuscript.

## Supplementary Material

Additional file 1 **Supplemental information**. All additional information regarding the article is described in detail in this PDF-document. Click here for file

Additional file 1 **Processed data in mascot generic file format**. Raw data of the described QTof measurements are converted to Mascot generic file format (mgf) for processing with CompNovo, PepNovo, and Peaks online. Files are zipped. Click here for file
