# Supplementary material for: Identification of a novel Plasmopara halstedii elicitor protein combining de novo peptide sequencing algorithms and RACE-PCR
Source: Proteome Sci. 2010 May 10;8:24. doi: 10.1186/1477-5956-8-24 (PMC2881003; doi:10.1186/1477-5956-8-24)
Supplement: Additional file 1 — Supplemental information. All additional information regarding the article is described in detail in this PDF-document. [file 1477-5956-8-24-S1.PDF]

Supplemental information

Content

Performance of de novo sequencing algorithms 2
Materials 3
De Novo algorithm parameters 3
Conversion to Mascot generic file 3
OpenMS 1.6 4
CompNovo 0.9 5
PEAKS online 2.0 6
PepNovo 7
Evaluation of peptide prediction 8
Isobaric amino acids 9
Peptide overview of data analysis 10
P. halstedii peptide identification with Mascot 13
P. halstedii protein and peptide predictions (*de novo*) 15
P. halstedii protein identification with BLAST and MS Blast 20
Additional References 22

Performance of de novo sequencing algorithms

Tab. S1: Performance of *de novo* sequencing algorithms.
The table summarizes performance comparisons of *de novo* sequencing algorithms of published data. Numbers in table represents prediction accuracy (highest numbers are highlighted).

de novo algorithm	DiMaggio & Floudas, 2007	Bertsch *et al*, 2009	Pitzer *et al.*, 2007	Ma *et al.*, 2003	Pevtsov *et al.*, 2006
Lutefisk	0.400	0.024	0.275	0.500	0.72
PepNovo	0.480	0.183	**0.298**	-	0.59
PEAKS	0.640	-	-	**0.944**	**0.80**
NovoHMM	-	-	-	-	0.15
EigenMS	0.640	-	-	-	-
CompNovo	-	**0.318**	-	-	-
AUDENS	-	-	-	-	0.03
PILOT	**0.720**	-	-	-	-
correct subsequence of length at least x	10	5 (a)	6	6	10 (b)
number of spectra peptides	25 (c)	2406	771	54	1405

(a) allowance of 3 amino acids substitution
(b) allowance of 4 amino acids substitution
(c) dataset of Ma *et al.* [1]

Materials

Tab. S2: Protein amount of samples for *de novo* sequencing test data set.

Sample	Protein amount
BSA	500fmol
Casein	1pmol
Cytochrome C	1pmol
Proteinmix (BSA, Lactate Dehydrogenase A, Hemoglobine A + B)	300fmol
Waters Mix (BSA, Enolase 1, Alcohol Dehydrogenase 1, Glycogen phosphorylase)	250fmol each

De Novo algorithm parameters

Conversion to Mascot generic file

For protein identification via Mascot database searching and also *de novo* sequencing, raw data was converted to Mascot generic file format (mgf) with the mascot.dll provided by the Analyst software (fig. S1). The default precursor charge state was selected to be two or three. The converter allows merging of single spectra. Since the converter seems to handle the merging options not exactly to the parameters given by the user, we exclude such possibility by setting the maximum number of cycles between groups for merging to zero. MS/MS data was processed by removing background peaks with a lower intensity of 0.5% of the highest intensity. All MS/MS data was centroid and de-isotoped, spectra with less than ten peaks were rejected.

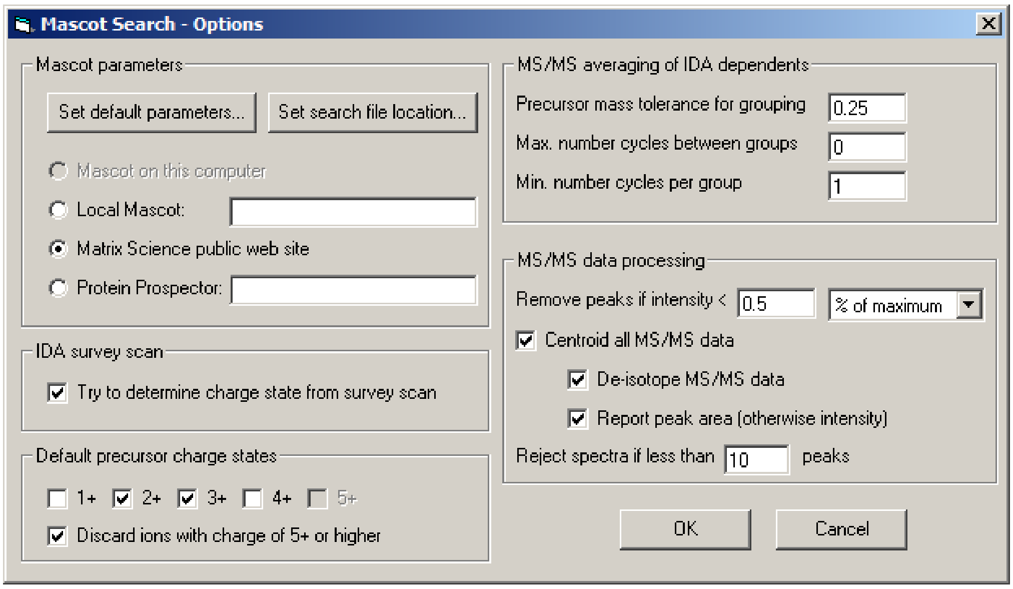

Fig. S1: Options of mascot.dll

OpenMS 1.6

OpenMS 1.6 proteomics pipeline tools (www.open-ms.de; TOPP and TOPPAS) were used for peptide identification with Mascot database search algorithm (version 2.2) and false-discovery rate evaluation. If not written elsewhere, standard settings were used for all OpenMS tools. All settings are explicitly explained in OpenMS help.

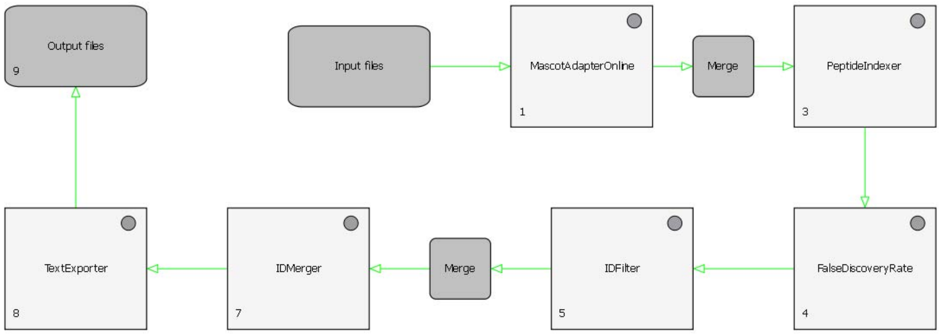

Fig. S2: TOPPAS pipeline for Mascot protein identification with false-discovery rate evaluation

In the first step the MascotAdapterOnline was used. A database consisting of the proteome of *Sorangium cellulosum* (9320 proteins), alcohol dehydrogenase 1 (yeast), cytochrome C (horse), glycogen phosphorylase b (rabbit), enolase 1 (yeast), BSA (bovine), hemoglobin subunit a and subunit b (bovine), L-lactate dehydrogenase (rabbit), alpha casein 1 and 2 (bovine), and typical contaminations like keratins and trypsin. The database also contained reversed sequences of all mentioned proteins for evaluating a false discovery rate. The following parameters were applied: One missed cleavage was allowed (with enzyme trypsin); precursor mass and product ion tolerance were set to 0.3Da; fixed modification of Cystein to Carbamidomethyl (+57.02146Da) and variable oxidation of Methionine (+15.9994Da), and only doubly and triply charged ions were selected.
In the next step all peptides were indexed according to the used database and decoy sequence matches were written as "_rev".
False discovery rate (FDR) was calculated using the FDR toolbox and peptides were filtered in the IDFilter. With these tools we discarded all peptides which are not matching with one of the proteins of the test set (BSA, alpha casein 1 and 2, cytochrome C, L-lactate dehydrogenase A, hemoglobine A + B, enolase 1, alcohol dehydrogenase 1, glycogen phosphorylase (tab. S2)). P-value was set to 0.05 and FDR to 0.01 with a minimum peptide length of 6 amino acids.
All peptide IDs were merged (IDMerger) and converted into human readable format with the TextExporter tool. All Mascot database options including the FDR are described in detail in Bertsch *et al*. [2].

CompNovo 0.9

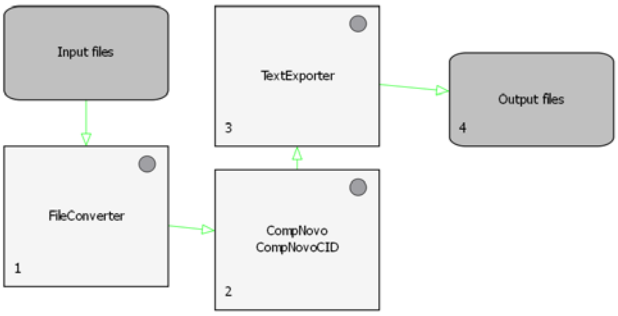

Fig. S3:TOPPAS pipeline for de novo sequencing using CompNovo

The *de novo* algorithm CompNovo is a module of OpenMS. CompNovo uses a divide-and-conquer approach, where the candidates are generated from subspectra via exhaustive enumeration of amino acid combinations [2]. All settings are explicitly explained in OpenMS help as well as in [2].

For *de novo* sequencing with CompNovo we generated a TOPPAS pipeline as depictured in fig. S). In the first step, the FileConverter toolbox converted all mgf files into mzML format because CompNovo as part of the multiplatform OpenMS pipeline works only with this universal file format. *De novo* sequencing was performed using CompNovoCID. Therefore, we applied the following parameters: maximal number of amino acids per decomp was set to 4; only tryptic peptides were sequenced; precursor mass and product ion tolerance were set to 0.3Da; max_number_pivot was set to 9; max_subscore_number was set to 40; double_charged_iso_threshold was set to 6; the m/z range included all peptides with a m/z between 200 and 2000; Max_isotope_to_score was set to 3; max_decomp_weight was set to 450; number_of_prescoring_hits was set to 250; modification of Cystein to Carbamidomethyl (+57.02146Da) and variable oxidation of Methionine (+15.9994Da). The output was exported into human readable text format (.txt).

PEAKS online 2.0

The freely-available software PEAKS online 2.0 (available at http://www.bioinformaticssolutions.com/peaksonline) is based on a dynamic programming algorithm which computes peptides whose ions correspond to as many high abundance peaks in the spectrum as possible. A reward or penalty score is computed for every possible mass value to achieve sequence identification even in the absence of peaks. Additional factors such as peak intensity rank of the peak and coexistence of other peaks, and mass errors improve the accuracy of the algorithm [1]. Data was uploaded as a Mascot generic file. Merge tolerance for MS/MS spectra was set to 0.3Da. Pre-processing and filter option were deactivated.

For PEAKS online 2.0, mgf files were uploaded on the web server and the following settings were used for *de novo* prediction of the QSTAR data (fig. S4). Trypsin was selected as endopeptidase. Carbamidomethylated cysteines were selected as fixed modification and oxidation of methionines (+15.99491Da) as variable modification. Tolerance for merging spectra was selected within m/z of 0.3Da. Parent tolerance and fragment tolerance was set to 0.3Da. We allowed up to three modifications per peptide. Results contained only one prediction per peptide.

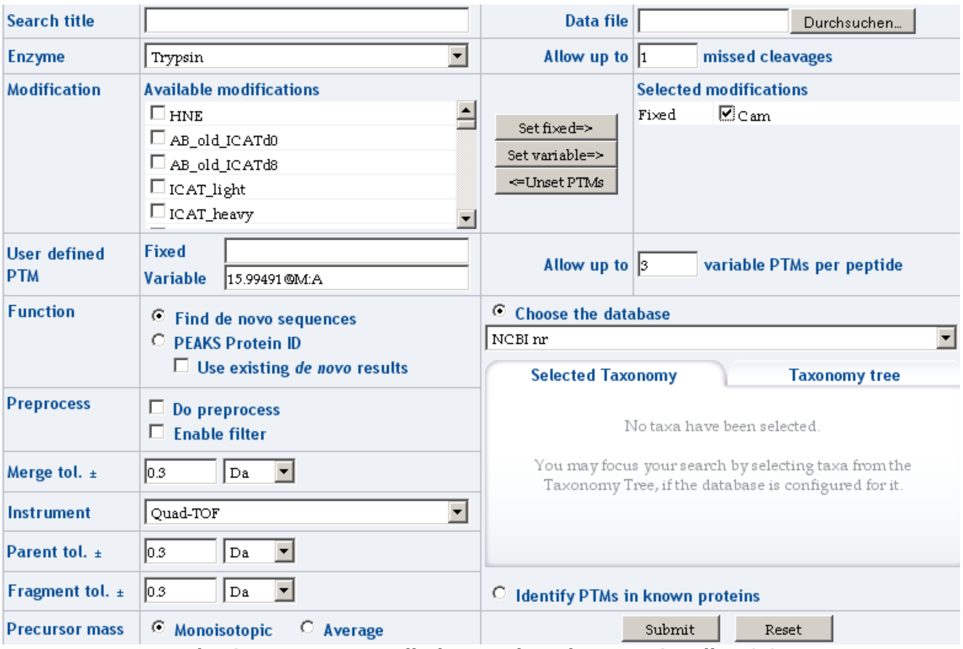

Fig. S4: *De novo* prediction settings in PEAKS online 2.0

Formatiert: Schriftart: Kursiv

PepNovo

PepNovo is an open-source algorithm in which each peak in a spectrum corresponds to a vertex in the graph. Two vertices are connected by an edge if the mass difference between them equals the mass of one or several amino acids. Vertices corresponding to N- and C-termini are usually added to the graph. The algorithm determines a path from the N- to C-terminus by generating a continuous sequence. The probabilistic network scoring is matched on random peak hypothesis and collision-induced dissociation hypothesis which determine the probability that the peaks in the spectra are randomly caused [3]. The PepNovo web server (version 2009-09-28) was used for this study (available at http://proteomics.bioprojects.org/MassSpec). Data was uploaded as a Mascot generic file.

For PepNovo (version 2009-09-28), mgf files were uploaded on the web server (fig. S5). The parent mass tolerance and fragment ion tolerance of 0.3Da were used for *de novo* prediction of QSTAR data. Trypsin was selected as endopeptidase. Carbamidomethylated cysteines were selected as fixed modification and as optional modification oxidation of methionines (+15.9994 Da). We allowed up to three modifications per peptide. Results contained only one prediction per peptide.

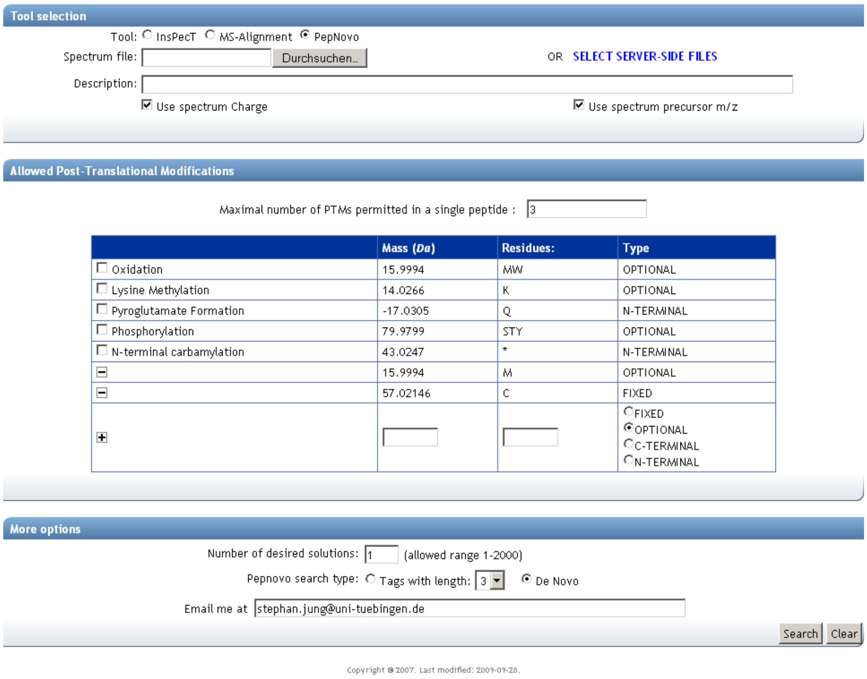

Fig. S5: ***De novo*** **prediction settings in PepNovo (version 2009-09-28)**

Evaluation of peptide prediction

Each *de novo* implication returns a list of more than one possible candidate for a given m/z and scan with different probabilities. We only evaluated the predicted peptide for each algorithm with the highest score and sequence prediction with a lower score for the same m/z and scan were ignored.

For example, the peptide VGGHAAEYGAEALER of HBA_BOVIN with an m/z of 510.574628 and a charge of z=3 was predicted by PEAKS online 2.0 as VTYEEYGAEALER with a score of 91, as VAAHNEYGAEALER with a score of 10 and also as VHAANEYGAEALER with a score of 10. For our study, we only used the sequence VGGHAAEYGAEALER, which was ranked with the highest score and ignored other predictions although there was a large overlap in sequence composition.

The PepNovo algorithm reports sequences with a gap because the spectrum did not provide enough information about amino acid composition or permuted amino acid sequences. We only evaluated continuous peptide sequences and neglected parts with unclear composition. For example, The same peptide as described above was reported in PepNovo as [522.201]VYGAEALER. The N-terminal gap of mass [522.201] could be due to low spectrum quality and would lead to unclear sequences. We did not further investigate such gaps and only included the predicted amino acid sequence VYGAEALER for our purpose which resulted in eight correctly predicted amino acids.

The same peptide candidate was also analysed with CompNovo. CompNovo was not able to annotate an amino acid sequence to this peptide. Recently, it was shown that sequence coverage decreased significantly in the CID spectra of triply charged peptides predicted by CompNovo [2].

Also, we did give credit to the algorithm concept for amino acid ambiguities by allowing up to three incorrect amino acid assignments per peptide.

Some proteins are known for their post-translational modifications (PTMs) like phosphorylation. We did not investigate the performance of the algorithms on PTMs beside the artificial modifications of carbamidomethylated cysteines and oxidized methionines which are introduced by the digestion step. Such an approach would exceed the scope of our study. Besides, our study showed clearly, that all tested *de novo* algorithms failed to reach at least a 50% threshold of exact peptide sequence identification. We expect that the identification rate would decrease dramatically for each additional PTM in *de novo* sequencing because of higher variance and no fitting sequences would be predicted in such cases.

Isobaric amino acids

Tab. S3: Isobaric amino acids.
Some amino acids cannot be distinguished with mass spectrometers because of their low resolution and mass accuracy which result in incorrect amino acid assignment of one residue by *de novo* sequencing algorithms by groups of isobaric amino acids.

Amino acids	Da (mono)	Amino acids	Da (mono)	delta mass
Lys (K)	128.09496	Gln (Q)	128.05858	0.03638
Ile (I)	113.08406	Leu (L)	113.08406	0
Phe (F)	147.06841	M^{Ox}	147.03540	0.03301

Peptide overview of data analysis

Tab. S4: Peptide overview of QSTAR test data set analysis.

The first column contains all measured peptides annotated by Mascot from mixtures of different standards. The following columns display *de novo* sequenced peptides and the associated scores of the different algorithms. A minimum of three consecutive correct amino acid assignment per peptide was marked in capital letter, oxidation of methionine is shown as M^{Ox}, and C^{CAMe} represents carbamidomethylation of cysteine. All amino acids are written in one letter code. In some cases, peptides were not found in CompNovo 0.9 and are marked as 'not found'.

Peptides	CompNovo 0.9	Score	PEAKS Online 2.0	Score	PepNovo	Score
SLGKVGTR	c^{CAMe}ftyvk	0.066	SLGKVdak	10	SLGQrtr	30
VDPVNFK	VDPVNFK	0.225	VDPVNFK	52	dvppfsr	39
MLTAEEK	MLTAEEK	0.197	MLTAEEK	99	MLTAEEK	94
FKDLGEEHFK	not found	----	ksgtGEEglpfk	10	m^{Ox}qfheglpfk	60
LSQKFPK	lfyynk	0.060	LSQKFPK	71	LSQKM^{Ox}PK	63
EC^{CAMe}C^{CAMe}DKPLLEK	c^{CAMe}dssnerqapk	0.206	dc^{CAMe}ssnePLLEK	10	eaanglhlpLEK	61
VKVDEVGGEALGR	grmpEVGGEALGR	1.033	ttgreggvepLGR	10	[214.097]dlgaVGGEALGR	139
DDSPDLPK	DDSPDLPK	0.712	DDSPDLPK	100	DDSPDLPK	80
LC^{CAMe}VLHEK	LC^{CAMe}VLHEK	0.158	LC^{CAMe}VLHEK	99	swVLHEK	73
VTLTSEEEAHLK	tlvtdvvpledr	0.121	tvLTSEEEAHLK	31	VTLTSEEEAHLK	109
IETMREK	LETMREK	0.054	LETMatlk	10	LETac^{CAMe}kk	27
LVIITAGAR	LVLLTAqr	0.080	LVLLTAqr	100	LVLLTAK[175.115]	63
ISGFPKNR	lswekqk	0.103	LSGFhttr	19	LSGM^{Ox}httr	45
AEFVEVTK	AEFVEVTK	0.143	AEFVEVTK	80	[101.044]vM^{Ox}VEVTK	85
YLYEIAR	YLYELAR	0.298	YLYELAR	100	YLYELAR	69
FIIPNVVK	FLLPvvnk	0.411	FLLPNVVK	50	FLLPhfr	46
AAVTAFWGK	AAVTAFWGK	0.675	AAVTAFWGK	100	[142.071]VTAFWGK	59
TC^{CAMe}VADESHAGC^{CAMe}EK	m^{Ox}nVADESHAGssdk	0.052	TC^{CAMe}VADESHAGC^{CAMe}EK	16	nm^{Ox}vgeESHAGttsk	67
IVSGKDYSVTANSK	not found	----	dpSGKDYSVTANSK	20	lvnyesgSVTANSK	50
DYSVTANSK	dywTANSK	0.943	DYSVTANSK	87	ysdvc^{CAMe}kr	30
TPVSEKVTK	ptvsc^{CAMe}vptk	0.063	TPVSc^{CAMe}pVTK	10	[198.097]VSEQVTK	21
VTLTSEEEAHLKK	aeLTSEmhptLKK	0.053	eaLTSEEEAHLKK	77	VTLTSEEEAHLQK	123
ALKAWSVAR	ALKAdawvk	0.094	ALKAWSVAR	49	algaasv[432.254]	48
NRVIGSGC^{CAMe}NLDSAR	not found	----	vvgnLGSGC^{CAMe}NLDSAR	10	[643.37]eaNLDSAR	76
QTALVELLK	QTALVELLK	0.148	QTALVELLK	40	QTALVELLK	96
VGGHAAEYGAEALER	not found	----	vtyeeYGAEALER	91	[522.201]vYGAEALER	117
LKEC^{CAMe}C^{CAMe}DKPLLEK	not found	----	knggpgsEC^{CAMe}C^{CAMe}pLEK	10	LQEedsgqeqLEK	48
VHPISTMLK	vhynlhsr	0.251	VHPLSTMLK	66	VHPLSTser	47
LC^{CAMe}VLHEKTPVSEK	not found	----	kfssgdrgaaam^{Ox}vvk	10	[751.347]ktltavr	35
QQEGESRLNLVQR	not found	----	apaegsdskkaLVQR	22	kqvdllhhaLVKR	47
VHPISTM^{Ox}LK	vhqrnM^{Ox}LK	0.146	hvPLSTM^{Ox}LK	32	VHPLSTpyk	45

QNC^{CAMe}DQFEK	KNC^{CAMe}enFEK	0.558	KNC^{CAMe}Dfgaek	14	elqffga[276.152]	41
FRYLMGER	qm^{Ox}flm^{Ox}lek	0.685	fmlsglem^{Ox}k	10	[131.045]flsfgltr	27
SHC^{CAMe}IAEVEK	hsC^{CAMe}LAEVEK	0.188	SHC^{CAMe}LAEVEK	31	[275.185]vealplr	49
LRVDPVNFK	hrlhhlfk	0.340	elklvpNFK	10	[384.043]vpvggfk	18
VLDSFSNGMK	VLDSFSNc^{CAMe}r	0.329	VLDSFSNGMK	100	lvDSFSNGMK	77
VDEVGGEALGR	VDEVGwspgr	0.251	VDEVGGEALGR	67	VDEVGGE[416.26]	70
VLSAADKGNVK	VLSnhhapvk	0.049	VLSAADKGNVK	35	VLSAADKGNVK	94
SADTLWGIQK	SADTLWGLQK	0.175	SADTLWGLQK	91	[158.065]DTLWGLQK	121
C^{CAMe}C^{CAMe}TESLVNR	C^{CAMe}C^{CAMe}TESLVNR	0.400	C^{CAMe}C^{CAMe}TESLVNR	99	[292.045]eESLVNR	64
LVNELTEFAK	pdNELhsshk	0.456	LVNELhsshk	35	hqslele[327.225]	8
RVHPISTMLK	qmmlsgm^{Ox}vlr	0.078	sslvfgslMLK	10	[390.218]stsevlr	27
DTHKSEIAHR	ehskdtwyk	0.086	DTHKdtLAHR	49	htdgadg[539.783]	54
C^{CAMe}ASIQKFGER	c^{CAMe}akthaahsgr	0.156	C^{CAMe}ASLQKFwr	78	[318.094]LKKFegr	88
KSADTLWGIQK	KSAtdevaavqk	0.257	KSAtdlwvaqk	10	QSAtdeglavkk	67
VIGSGC^{CAMe}NLDSAR	VLGSGC^{CAMe}NLDSAR	0.381	VLGSGC^{CAMe}NLDSAR	99	VLGSc^{CAMe}nl[448.221]	78
QVVDSAYEVIK	lnVDSAYEVLK	0.313	QVVDSAYEVLK	21	[227.128]VDSAYEVLK	83
HLVDEPQNLIK	HLVDststnfgk	0.495	HLVDEqpNLLK	17	lhVDEPelLLK	85
ETYGDMADC^{CAMe}C^{CAMe}EK	teYGDMADC^{CAMe}nm^{Ox}r	0.537	ETYGDMADyggdgk	18	ETYGDMAy[548.209]	54
EYEATLEEC^{CAMe}C^{CAMe}AK	yeEATLEEC^{CAMe}C^{CAMe}AK	0.377	EYEATLEhgptgak	83	EYEATLEE[538.179]	65
DLGEEHFK	veGEEHFK	0.094	nnGEEHFK	32	nnGEEHFK	57
NEC^{CAMe}FLSHK	NEC^{CAMe}FLSHK	0.176	qdC^{CAMe}FLSHK	21	ggessvn[404.068]	44
KVPQVSTPTLVEVSR	not found	----	lggvpagSTPTLVEVSR	28	[450.169]tSTPTLVEVSR	103
EAC^{CAMe}FAVEGPK	swsFAVEGPK	0.860	vtC^{CAMe}FAVEGPK	46	[360.041]fadlGPK	29
KQTALVELLK	qedmhELLK	0.151	KQTlaVELLK	10	qqqqqel[260.169]	45
EC^{CAMe}C^{CAMe}HGDLLEC^{CAMe}ADDR	not found	----	qm^{Ox}ynryLEC^{CAMe}ADDR	15	[635.223]elllavADDR	83
FKDLGEEHFK	M^{Ox}KDLwEHM^{Ox}K	0.106	FKDLGEEHFK	31	FQDLwEHFK	98
EC^{CAMe}C^{CAMe}DKPLLEK	c^{CAMe}eC^{CAMe}DKPLLEK	0.124	EC^{CAMe}C^{CAMe}DKPLLEK	65	[448.931]DKPLLEK	98
GAC^{CAMe}LLPK	qdlaspk	0.103	agC^{CAMe}LLPK	10	AC^{CAMe}LLPK	69
DDPHAC^{CAMe}YSTVFDK	dlvhagyyqVM^{Ox}DK	0.088	etPHAvhsSTVFDK	17	[230.033]PHAlqpaTVFDK	86
LKPDPNTLC^{CAMe}DEFK	LKPDnppfdvc^{CAMe}fk	0.372	algpdnptlqvavpgk	10	lqlvnptlqfEFK	44
EDVPSER	devpm^{Ox}pk	0.913	mlVPSER	17	[128.046]gaspfpk	-2
NMAINPSK	NMALqaar	0.830	NMALNlak	84	NMALNPSK	71
AMKPWIQPK	nesdkppspk	0.235	sdQPWLQPK	10	sdqpsvLKPK	59
AM^{Ox}KPWIQPK	AFQdkshgpk	0.369	AFQdkqlhk	10	[218.068]qplwKPK	54
NAVPITPTLNR	NAVPLTgsathk	0.062	gqVPLTgsgshr	51	gkVPLTsg[456.257]	46
ALNEINQFYQK	spggELNQFYQK	0.969	ALNELNQFYKK	38	ALNELNQFYQK	90
M^{Ox}IFAGIKK	pyFAGLKK	1.258	M^{Ox}LFAGLQK	10	lfeavc^{CAMe}k	18
EDLIAYLK	EDLLAYLK	1.089	EDLLAYLK	98	[129.068]DLLAYLK	46
KTGQAPGFTYTDANK	not found	----	wwlPGFTYTDANK	33	addlAPGFTYTDANK	102

TGQAPGFTYTDANK	atnadwrvaqgpgk	0.750	TGKAPGFytswqk	10	gldawdt[711.391]	15
IGDYAGIK	LGDYqlk	0.163	LGDYAGLK	74	glDYAGLK	68
MSLVEEGAVK	MSLVEEqvk	0.219	MSLVEEqvk	68	MSLVEhf[219.077]	70
VAAAFPGDVDR	VAAAM^{Ox}PtaVDR	0.103	VAAAFPeaadr	77	avAAM^{Ox}Peawr	89
GLAGVENVTELK	GLAGVENVTELK	0.556	vaAGVENVTELK	66	[170.102]AGVENVTELK	106
SISIVGSYVGNR	SLSLVGSYVGNR	0.265	SLSLVGSYVGNR	25	lsSLVGS[608.313]	62
TC^{CAMe}VADESHAGC^{CAMe}EK	c^{CAMe}tVADESHAac^{CAMe}dk	0.378	c^{CAMe}tVADESHAGC^{CAMe}EK	53	[261.076]VADESHAC^{CAMe}EK	89
ETYGDM^{Ox}ADC^{CAMe}C^{CAMe}EK	teYGDM^{Ox}ADC^{CAMe}C^{CAMe}EK	0.536	teYGDFADC^{CAMe}C^{CAMe}EK	79	ETYGDFADy[433.166]	55

Tab. S5: Correctly predicted amino acids for the protein test data set.
The table shows the total number of amino acids predicted by each algorithm. The third row contains the number of correctly predicted amino acids for each algorithm. The average peptide length was calculated by dividing the total number of amino acids through the number of peptides. The average rate of correct amino acid prediction was calculated by dividing the correctly predicted amino acids through the total of amino acids.

	Mascot	CompNovo 0.9	PEAKS online	PepNovo
total amino acids	796	653	812	678
correctly predicted amino acids	-----	354	573	401
average peptide length	10	8	10	9
average rate of correct amino acid prediction per peptide	----	54%	71%	59%

P. halstedii peptide identification with Mascot

Automatically Mascot database searching of *P. halstedii* MS data resulted in five proteins (tab. S6). Four of these five proteins were only identified by one peptide. These proteins are called “one-hit-wonder” and are not reliable for solid scientific research. The first protein is identified by two peptides.

Fig. S6 shows that the homology from the house-keeping protein cytochrome oxydase II is closer to the human analog than to the bacterial protein. In our database searching of MS data we identified only bacterial proteins (tab. S6). We assumed same relationship for other proteins and excluded bacterial contamination by multiple sequencing of protein and DNA from different *P. halstedii* strains. Therefore we could not rely on the identification from the Mascot database searching and initiated the *de novo* peptide sequencing.

Tab. S6: Results of automatically Mascot database searching using Swissprot database (all organisms).
The table shows the results of the search using Mascot and Swissprot database. The first and the second column contain information about the located protein and the organism. m/z, peptide sequence and corresponding Mascot score are written in the third and fourth column. The consensus of the amino acids of the located peptide and the protein sequence of *P. halstedii* is defined in the last column.

protein name	Organism	m/z	Peptide	Mascot score	consensus
Phosphoenolpyrovate carboxykinase	Thermoanaerobacter tengcongensis	495.39	TTLSADPER	47	8/9 (88%)
		743.29	DGDTALFFGLSGTGK	43	15/15 (100%)
Phosphoenolpyrovate carboxykinase	Sulfurimonas denitrificans	425.67	MSIKDTR	61	7/7 (100%)
		433.66	M^{Ox}SIKDTR	32	6/7 (86%)
Phosphoenolpyrovate carboxykinase	Bacillus licheniformis	494.73	TTLSADPKR	54	9/9 (100%)
Surfactin synthetase subunit 3	Bacillus subtilis	425.68	YADLIQK	50	7/7 (100%)
50S ribosomal protein L6	Lactobacillus delbrueckii subsp. bulgaricus	504.21	YADEVVRR	19	3/8 (38%)

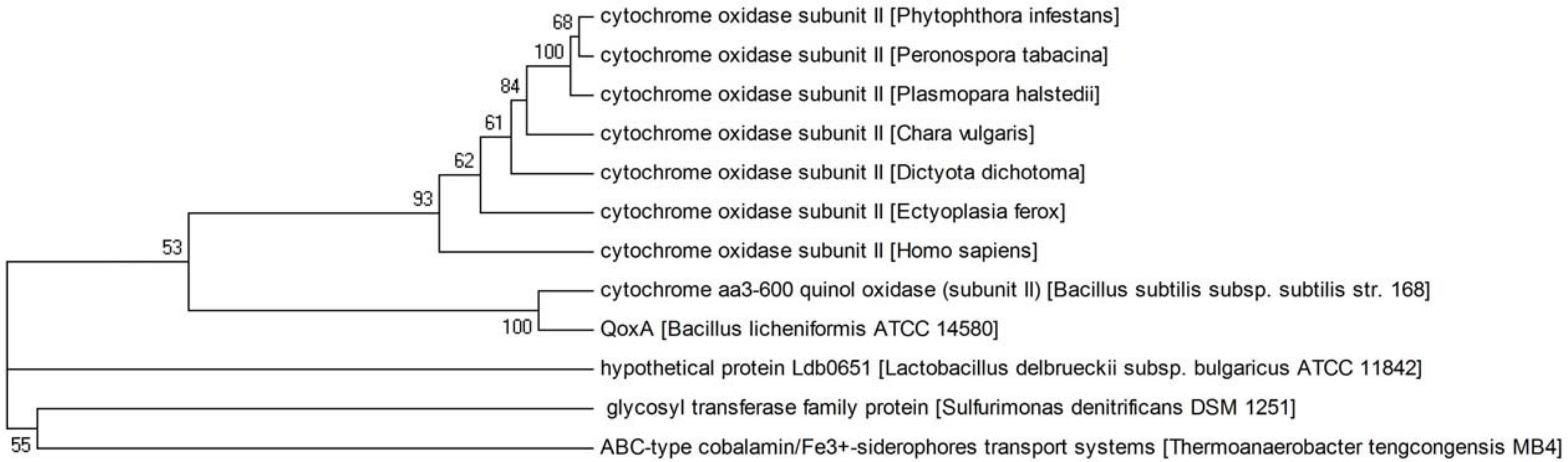

Fig. S6: Phylogenic tree based on cytochrome c oxydase II amino acid sequence of *P. halstedii* and other organisms.
The number of branches in the phylogenetic tree based on cox2 amino acid sequence data from *P. halstedii* indicate Bootstrap support in minimum evolution analyses. The sequence data was assembled and edited using the SeqMan module of the Lasergene software package, version 5 (DNAStar, Madison, WI). Alignments and phylogenetic reconstructions and minimum evolution phylogenetic inference were done using the default settings of MEGA (version 4) [4].

P. halstedii protein and peptide predictions (*de novo*)

All peptide candidates predicted by each algorithm were compared to the protein sequence of *P. halstedii* (fig. S7; tab. S7).

```
  1 CDLVSFSSHL NSPAKPPTIY RMLMAALRVS LRMQCIGSVK LMKSTHLLSS  50
 51 SFALRTFANA SASSLGLEKF GITNAKTQVH HNLSYDEIAA HEERNNEGQF 100
           QGA SASSLGLEK
            KK SASSLLVSK
           NAA SASSL
             A SASSLG
           NAA SASSLGLEK
           SPA SASSYHLK
101 IKNGTYTIDT GKFTGRSPKD KYIVDQAPSS KNIWWGDINH PVSAEVFDEL 150
      DGTYTLDT GK          YLVDEAPSS K
      NGTYTLDT GK        E NYLVDKAPSS K
                         D KYLVDEAPSS K
      DGTYTMQ              YLVDEAP
      NGTYTLD               LVDEANER
      DGTYTLDT GK          YLVDEASRR
      NGTYTNNTGK         D KYLVDPNYPK
151 YKTVTKHYGS AEKVYVFDGY AGAHAASRKK VRFITELAWQ HHFVTNMFLR 200
                                                    DVLVLY
201 PKTKDEIANF RPDFTIVNAC KVTNKSYKKH GLNSEVFVAF NIEKDVAVIG 250
    PK
251 GTWYGGEMKK GIFSMMNYWL PLDGIMAMHC SANKGKDGDT ALFFGLSGTG 300
                                          DDNDA LMRLGR
301 KTTLSADPQR YLIGDDEHGW DDEGIFNFEG GCYAKTINLS AENEPDIYNA 350
     TTLSADQPR
     TTLSADEPR
     TTLSADS
     TTLSADS
     TTLSADQPR
            EN YLVGGPNYPK
351 IKRDALLENV FVDTKTKIPD LYNTSKTENG RVSYPIHHIP NYEPTSSGGH 400
        WLLENV FVDTK  LPD FYNTSK
          LENV MTLTK  LPD MYNTSK
       NNALENV MVDTK                        TVD SWEPR
401 PSNVVFLTCD AYGVLPPVSK LSDGQAMYHF LSGYTAKVAG TERGVTEPTA 450
451 TFSACFGAAF LPLHPTKYAD LLQKKLQRHN TSVYLVNTGW TSGGYGVGKR 500
                  YAD LLQK
                  YAD LLKKK
                  YAD LLKK
                   AD LLQKK
                  YAN NLQK                PNFGT T----GVNK
                  YAD LLKQK
501 MSIKDTRACI DAILDGSIKK SEFTKDPNFG FEVPKRLGSI PENVLNPREA 550
           CAL DALGNGGSLK      NP-FG MEVPK LGSL PENVRAPR
           ACN DALLDVASK       DPNMG FEVPK LGSL PEDVRAPR
             L DALDLGD           NFG FEPVK LGSL PENVLN
             L DALLDGD           NFG MLDPK LGVV VEDVLNPR
           MTL DALVEGTAR                   LGSL PENQVVPR
551 WNDKDAYDAT AKKLAGMFKE NFRKYVSXGV TDYSKFGPKV LRLSNNVVYS 600
      DKDDTTMV PK
601 NVKDGIRRCL YMTVPGIKLI NDILEDKKKK K                     631
                         NRVVQDQVVP R
```

Fig. S7: Alignment of *P. halstedii* and all peptide candidates predicted by all three algorithms. Red highlighted peptides were predicted by PEAKS online, green by PepNovo and blue by CompNovo. All peptides were manually evaluated.

Tab. S7: Peptide candidates for *P. halstedii* protein predicted by each algorithm
The table contains the peptide candidates predicted by each algorithm. The position of each peptide is shown in accordance to its position to translated ORF of *P. halstedii* protein (numbers in brackets represent consensus to alignment in percent). The table is divided into three parts: (i) peptide candidates predicted by PEAKS online (red), (ii) peptides predicted by Pepnovo (green) and (iii) peptides predicted by CompNovo (blue). All peptides were manually evaluated.
Dark Grey marked rows present predicted sequences with a confidence score threshold 90>x>80 for either Peaks Online or PepNovo and their corresponding sequences in CompNovo. Light grey marked rows represent sequences already predicted before with a threshold above 90 for one of the *de* novo sequencing algorithms. Rows marked in lilac represent peptides which were automatically identified by Mascot database searching.

PEAKS online					
**m/z**	**z**	**predicted sequence**	**score**	**position in P.halstedii**	**correct matching amino acids**
425.7	2	YADLLQK	94	468 – 474	7/7 (100%)
494.7	2	TTLSADQPR	14	302 – 310	7/9 (78%)
517.7	2	NPFGM^{Ox}EVPK	37	526 – 535	8/10 (80%)
535.7	2	DGTYTLDTGK	99	103 – 112	9/10 (90%)
542.7	2	LPDFYNTSK	99	368 – 376	8/9 (89%)
554.7	2	YLVDEAPSSK	100	122 – 131	9/10 (90%)
575.2	2	DPNM^{Ox}GFEVPK	19	526 – 535	10/10 (100%)
638.3	2	C^{CAMe}ALDALGNGGSLK	34	508 – 518	8/13 (62%)
654.8	2	LGSLPENVRAPR	88	537 – 548	10/12 (83%)
682.3	2	WLLENVFVDTK	87	355 – 365	10/11 (91%)
495.4	2	TTLSADEPR	48	302 – 310	7/9 (78%)
574.2	2	QGASASSLGLEK	23	58 – 69	10/12 (83%)
574.5	2	KKSASSLLVSK	80	59 – 69	6/12 (50%)
655.3	2	LGSLPEDVRAPR	24	537 – 548	9/12 (75%)
675.8	2	ENYLVDKAPSSK	57	120 – 131	10/12 (83%)
535.2	2	NGTYTLDTGK	99	103 – 112	10/10 (100%)
638.8	2	AC^{CAMe}NDALLDVASK	22	508 – 519	8/12 (67%)
489.7	2	YADLLKKK	50	468 – 475	8/8 (100%)
676.3	2	DKYLVDEAPSSK	27	120 – 131	11/12 (92%)
**PepNovo**					
**m/z**	**z**	**predicted sequence**	**score**	**position in P.halstedii**	**correct matching amino acids**
425.7	2	YADLLKK	111	468 – 474	7/7 (100%)
494.7	2	TTLSADS[313.151]	98	302 – 308	6/7 (86%)
517.7	2	[96.894]NFGFEPVK	102	528 – 535	6/8 (75%)
535.7	2	DGTYTMQ[274.035]	95	103 – 109	4/7 (57%)
542.7	2	LPDM^{Ox}YNTSK	96	368 – 376	8/9 (89%)
554.7	2	YLVDEAP[321.163]	93	122 – 128	6/7 (86%)
575.2	2	[212.052]NFGM^{Ox}LDPK	90	528 – 535	6/8 (75%)
638.3	2	[231.052]LDALDLGD[232.049]	108	510 – 517	5/8 (63%)
654.8	2	LGSLPENVLN[272.154]	94	537 – 546	10/10 (100%)
682.3	2	[299.126]LENVM^{Ox}TLTK	96	357 – 365	7/9 (78%)
495.4	2	TTLSADS[314.461]	90	302 – 308	6/7 (86%)
574.2	2	NAASASSL[446.247]	89	58 – 65	6/8 (75%)
574.5	2	[185.497]ASASSLG[389.218]	89	60 – 66	7/7 (100%)
655.3	2	LGVVVEDVLNPR	88	537 – 548	8/12 (67%)
675.8	2	[404.021]DVLVLYPK	90	195 – 202	3/8 (38%)
535.2	2	NGTYTLD[305.171]	74	103 – 109	7/7 (100%)
638.8	2	[232.019]LDALLDGD[232.051]	94	510 – 517	7/8 (88%)
489.7	2	[163.040]ADLLQKK	110	469 – 475	7/7 (100%)
676.3	2	[406.168]LVDEANER	82	123 – 130	4/8 (50%)

Tab. S7, continued

CompNovo 0.9					
**m/z**	**z**	**predicted sequence**	**score**	**position in P.halstedii**	**correct matching amino acids**
425.7	2	YANNLQK	0.022	468 – 474	5/7 (71%)
494.7	2	TTLSADQPR	0.029	302 – 310	7/9 (78%)
517.7	2	PNM^{Ox}GTTGVNK	0.130	486 – 499	6/14 (43%)
535.7	2	DGTYTLDTGK	0.080	103 – 112	9/10 (90%)
542.7	2	DGTYTLDTGK	0.080	103 – 112	9/10 (90%)
554.7	2	YLVDEASRR	0.122	122 – 130	5/9 (56%)
575.2	2	DKDDTTMVPK	0.120	553 – 562	4/10 (40%)
638.3	2	DDNDALMRLGR	0.089	286 – 296	3/11 (27%)
654.8	2	LGSLPENQVVPR	0.055	537 – 548	9/12 (75%)
682.3	2	NNALENVM^{Ox}VDTK	0.118	354 – 365	9/12 (75%)
495.4	2	TVDSWEPR	0.025	388 – 395	2/8 (25%)
574.2	2	NAASASSLGLEK	0.044	58 – 69	10/12 (83%)
574.5	2	SPASASSYHLK	0.025	58 – 68	6/11 (55%)
655.3	2	NRVVQDQVVPR	0.060	621 – 631	3/11 (27%)
675.8	2	ENYLVGGPNYPK	0.217	309 – 320	3/12 (25%)
535.2	2	NGTYTNNTGK	0.140	103 – 112	8/10 (80%)
638.8	2	MTLDALVEGTAR	0.197	508 – 519	5/12 (42%)
489.7	2	YADLLKQK	0.141	468 – 475	8/8 (100%)
676.3	2	DKYLVDPNYPK	0.162	120 – 142	8/23 (35%)

Of all candidate peptides, we evaluated the average prediction of correct amino acids for each algorithm (total number of correct matching amino acids divided by total number of amino acids [%]): PEAKS online (83%), PepNovo (79%), CompNovo (56%).

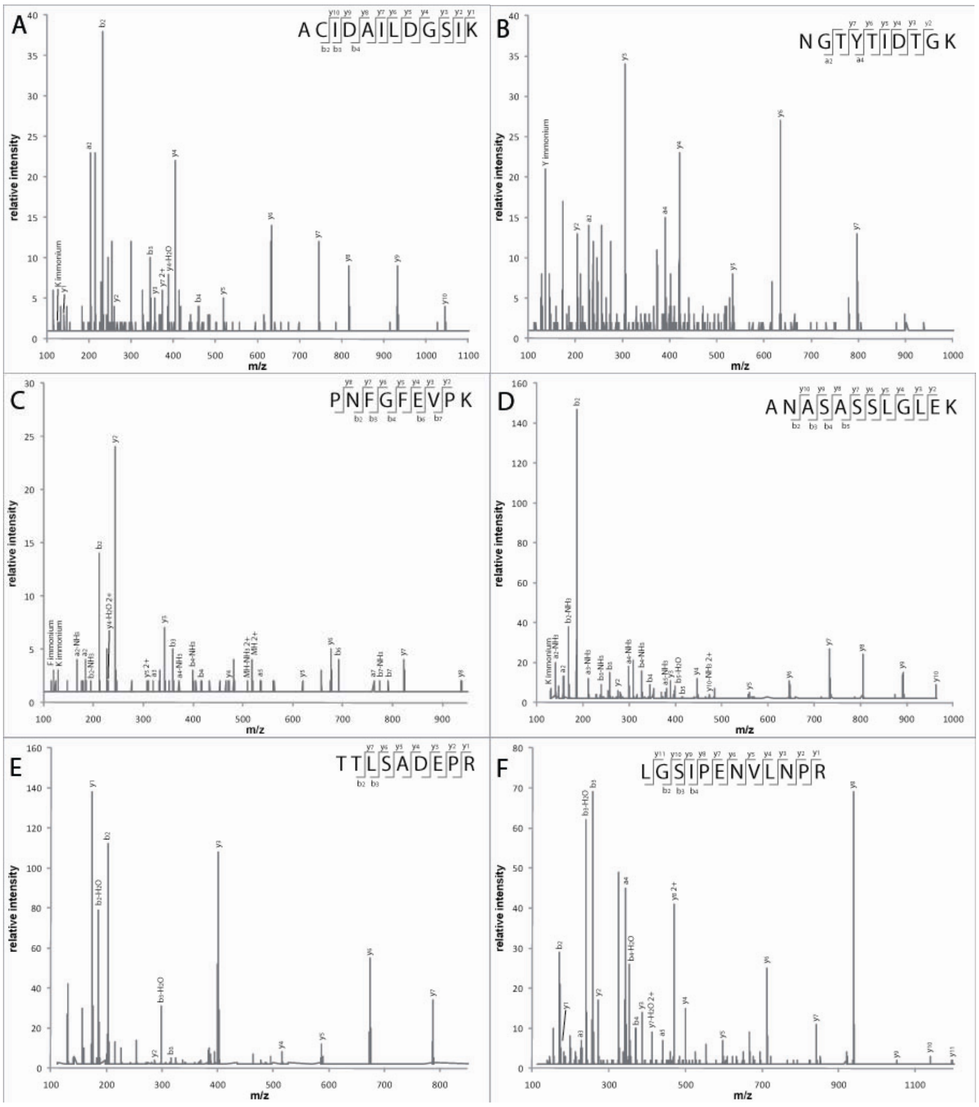

Fig. S8: MS/MS spectra of candidate peptides of 57kDa protein of *P. halstedii* sequenced by PEAKS online and PepNovo (threshold of 80).

The figure shows the MS/MS spectrum of the precursor ion at m/z 638.264 (A), 535.708 (B), 517.712(C), 574.245 (D), 495.398 (E), 654.797 (F), 542.720 (G), 574.5 (H), 655.283 (I), 682.269 (J), 676.256 (K), 494.726 (L), 425.677 (M), and 575.246 (N). The corresponding fragment ions are annotated and shown.

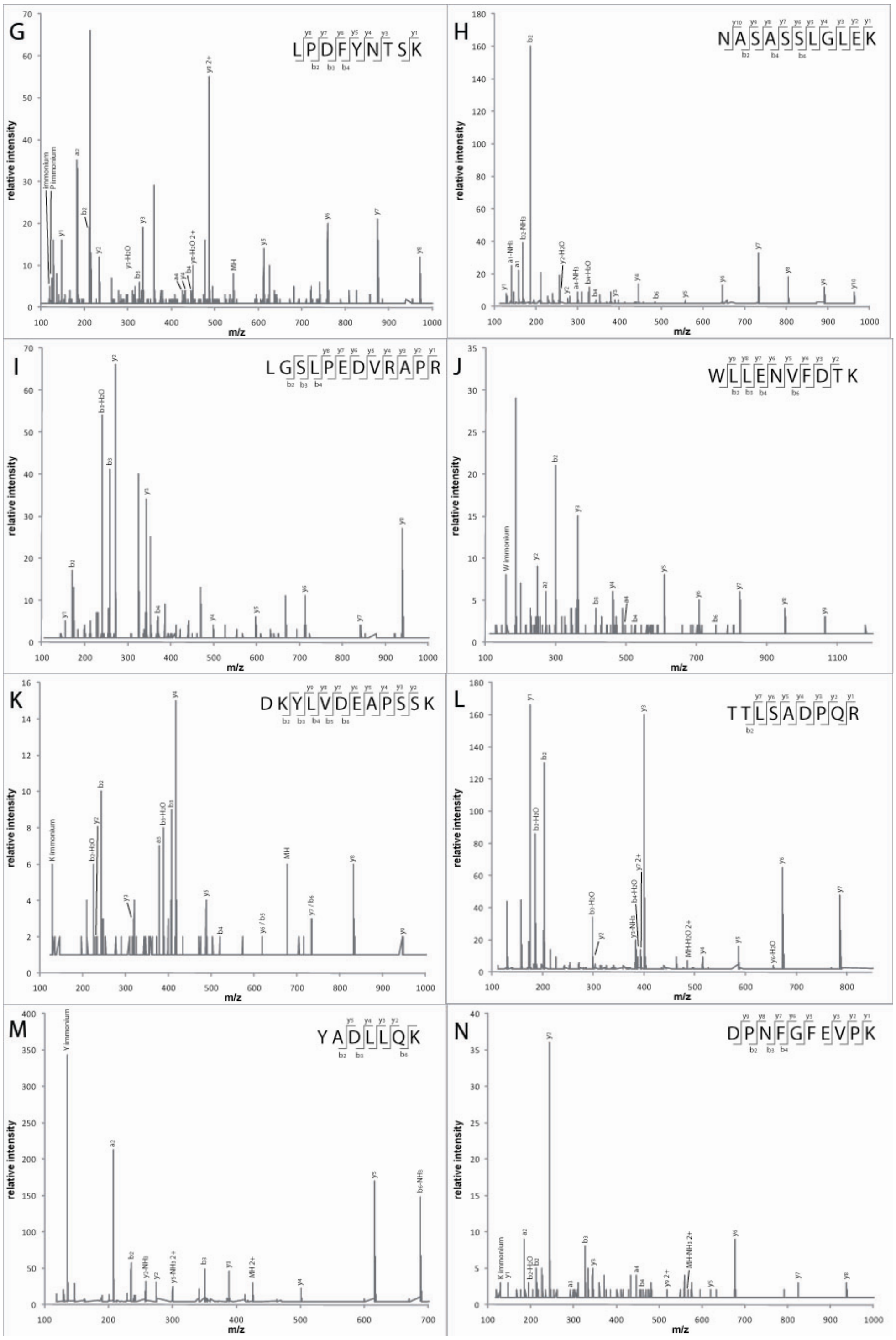

Fig. S8, continued

P. halstedii protein identification with BLAST and MS Blast

Tab. S8: Database searching of homolog peptides of all *de* novo candidates in BLAST.
All *de novo* sequenced peptide candidates were blasted against Swissprot to find homolog peptides to the query.

Subject id	% identity	alignment length	mis-matches	gap openings	q. start	q. end	s. start	s. end	e-value	bit score
gi|169838863|ref|ZP_02872051.1|	28.2	78	56	0	90	167	45	122	0.29	41.6
gi|71026205|ref|XP_762787.1|	34.0	50	33	0	94	143	1351	1400	0.4	41.2
gi|116489808|gb|ABJ98811.1|	36.2	47	30	0	68	114	237	283	1.7	32.2
gi|116489808|gb|ABJ98811.1|	37.5	24	15	0	117	140	289	312	1.7	24.5
gi|189474731|gb|ACE00054.1|	31.8	44	30	0	76	119	37	80	2.6	38.5
gi|146328651|ref|YP_001209254.1|	42.9	35	20	0	66	100	1405	1439	4.9	34.4
gi|146328651|ref|YP_001209254.1|	42.9	35	20	0	66	100	1524	1558	4.9	34.4
gi|146328651|ref|YP_001209254.1|	42.9	35	20	0	66	100	1643	1677	4.9	34.4
gi|146328651|ref|YP_001209254.1|	30.6	36	25	0	117	152	1530	1565	4.9	26.3
gi|146328651|ref|YP_001209254.1|	30.6	36	25	0	117	152	1649	1684	4.9	26.3
gi|146328651|ref|YP_001209254.1|	31.4	35	24	0	117	151	1768	1802	6.6	25.9
gi|189474775|gb|ACE00076.1|	29.6	44	31	0	76	119	37	80	9	36.7
gi|189474761|gb|ACE00069.1|	29.6	44	31	0	76	119	37	80	9	36.7
gi|189474729|gb|ACE00053.1|	29.6	44	31	0	76	119	37	80	9	36.7
gi|150397984|ref|YP_001328451.1|	33.3	51	34	0	110	160	352	402	9	36.7
gi|110799180|ref|YP_694610.1|	29.6	44	31	0	76	119	496	539	9	36.7

All peptide candidates were searched in BLAST after *de novo* sequencing as one protein against the NCBInr database to identify homolog proteins. As seen in tab. S8, no protein hit with a significant e-value or high percentage in identity could be found. The same search was also performed with the single peptide candidates but failed to produce any hit as the peptide length was too short to report any hits (data not shown).

Tab. S9: MS-BLAST database search of all candidate peptides of 57kDa protein of *P. halstedii*

m/z	*de novo* sequence	sequence homology by MS BLAST	Score	Organism
495.4	TTLSADQPR	TTLSADPTR	57	*B. melitensis* (bacteria)
575.2	DPNMGFEVPK	DPNFGFAVP	51	*B. melitensis* (bacteria)
682.3	WLLENVFVDTK	LENVVLD	35	*B. melitensis* (bacteria)
654.8	LGSLPENVRAPR	GSLTENTR	34	*B. melitensis* (bacteria)

All peptide candidates were searched in MS BLAST [5] using the standard settings after *de novo* sequencing as one protein against the swissprot database to identify homolog proteins. As seen in tab. S9 only partial sequence homology with low scores were found for bacterial proteins. The same search was also performed with the single peptide candidates but failed to produce any hit as the peptide length was too short to report any hits (data not shown).

Additional References

Reference List

1. Ma B, Zhang K, Hendrie C, Liang C, Li M, Doherty-Kirby A *et al*.: **PEAKS: powerful software for peptide de novo sequencing by tandem mass spectrometry.** *Rapid Commun Mass Spectrom* 2003, **17:** 2337-2342.

2. Bertsch A, Leinenbach A, Pervukhin A, Lubeck M, Hartmer R, Baessmann C *et al*.: **De novo peptide sequencing by tandem MS using complementary CID and electron transfer dissociation.** *Electrophoresis* 2009, **30:** 3736-3747.

3. Frank A, Pevzner P: **PepNovo: de novo peptide sequencing via probabilistic network modeling.** *Anal Chem* 2005, **77:** 964-973.

4. Tamura K, Dudley J, Nei M, Kumar S: **MEGA4: Molecular Evolutionary Genetics Analysis (MEGA) software version 4.0.** *Mol Biol Evol* 2007, **24:** 1596-1599.

5. Shevchenko A, Sunyaev S, Loboda A, Shevchenko A, Bork P, Ens W *et al*.: **Charting the proteomes of organisms with unsequenced genomes by MALDI-quadrupole time-of-flight mass spectrometry and BLAST homology searching.** *Anal Chem* 2001, **73:** 1917-1926.
